# Supplementary figures and images for: Computational modeling of the cephalic arch predicts hemodynamic profiles in patients with brachiocephalic fistula access receiving hemodialysis
Source: PLoS One. 2021 Jul 14;16(7):e0254016. doi: 10.1371/journal.pone.0254016 (PMC8279323; doi:10.1371/journal.pone.0254016)

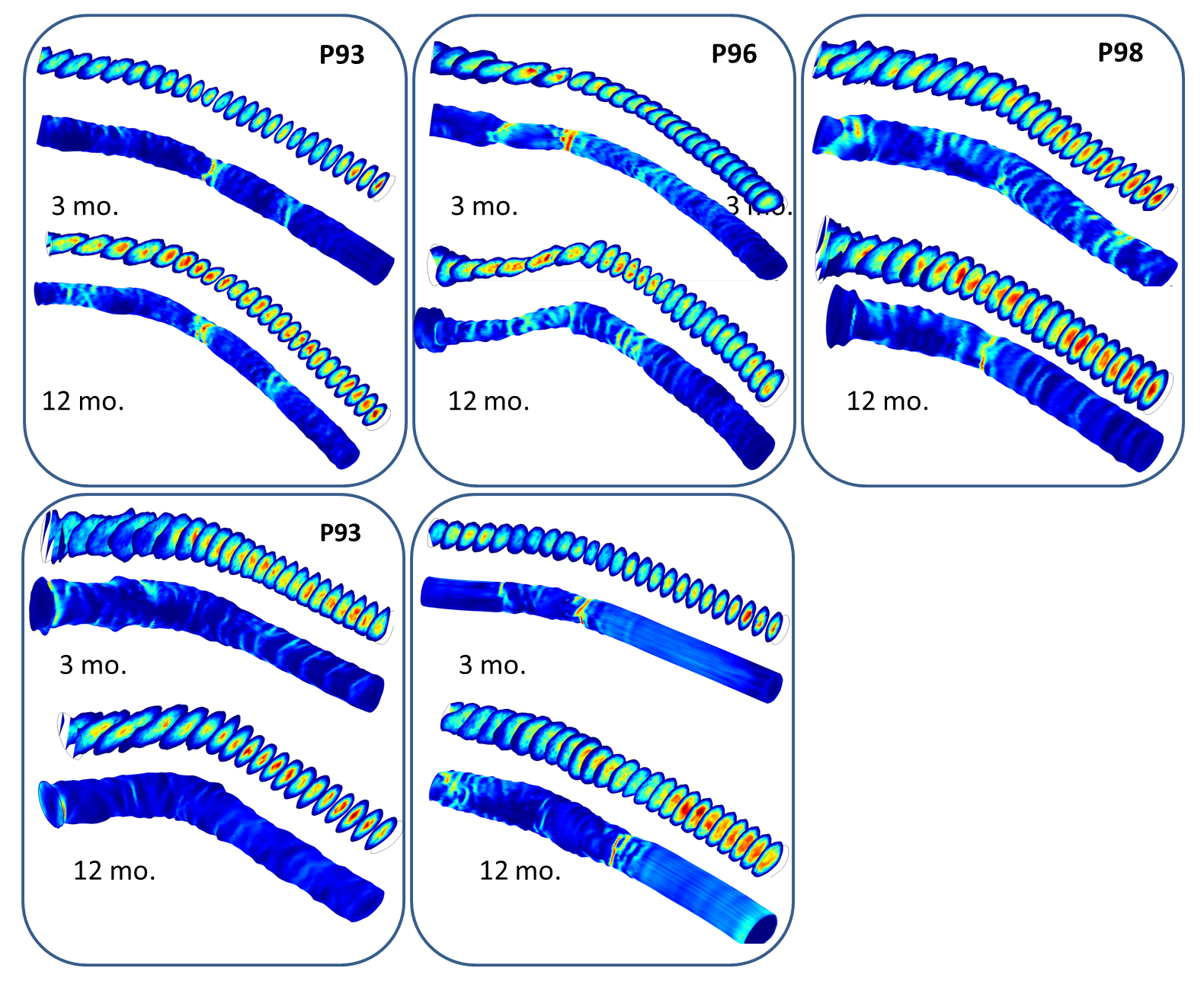

Supplement: S1 Fig — The color indicates the magnitude of Recell and WSS, respectively, following a rainbow scheme, with blue denoting the lowest and red the highest values. (TIF) [file pone.0254016.s011.tif]

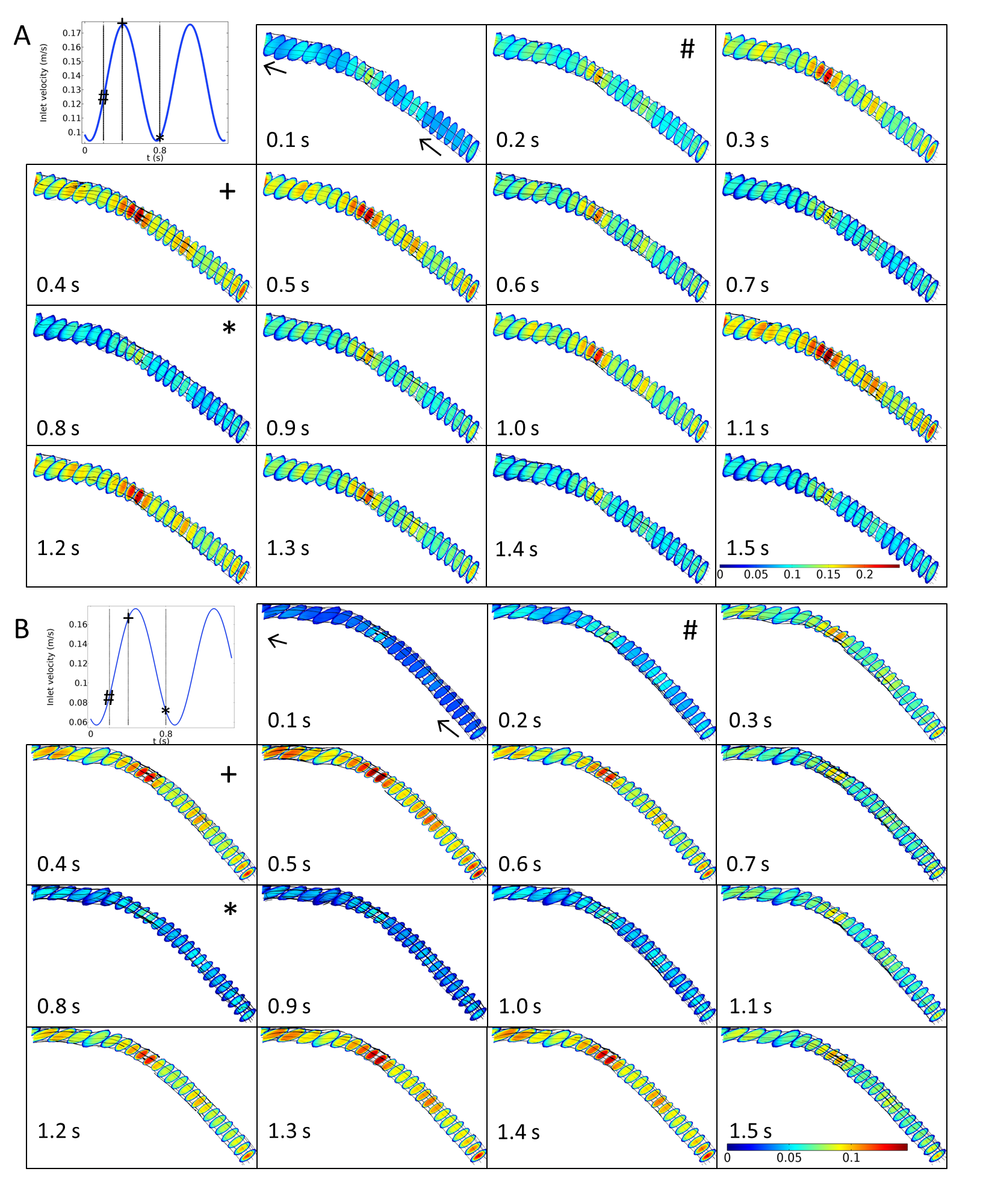

Supplement: S2 Fig — The velocity profile in the CA of patient P93 at (A) 3 months and (B) 12 months under pulsatile flow are shown at different times as indicated. The first panel in each figure shows the inlet velocity as it increases (systolic) and decreases (diastolic) as function of time. Inlet velocities and their corresponding velocity profiles are marked as ‘#’, ‘+’ and ‘*’ at three time-points, 0.2, 0.4 and 0.8 s. The color bar in the last panel indicates the lowest velocity in blue and the highest in red. Arrows in the second panel indicate the direction of flow along the CA. (TIF) [file pone.0254016.s012.tif]

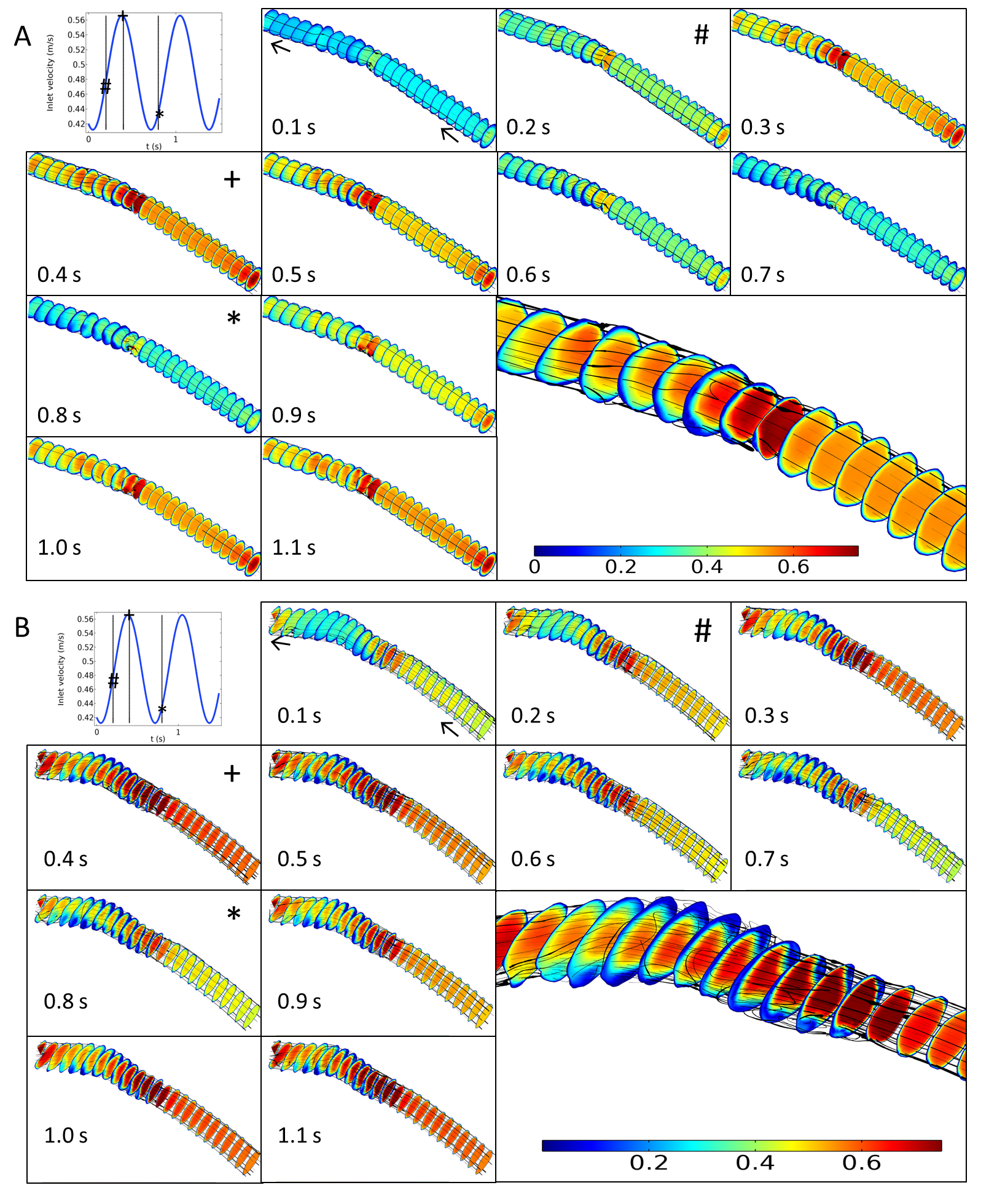

Supplement: S3 Fig — The velocity profiles in the CA of patient P122 at (A) 3 mo., and (B) 12 mo. under pulsatile flow are shown at different time-points. The first panel shows the inlet velocity as it increases (systolic) and decreases (diastolic) as function of time. Inlet velocities and their corresponding velocity profiles are highlighted at three time-points, 0.2, 0.4 and 0.8 s, marked as ‘#’, ‘+’ and ‘*’. The color bar in the last panel indicates the lowest velocity in blue and the highest in red. Arrows in the second panel indicate the direction of flow along the CA. (TIF) [file pone.0254016.s013.tif]

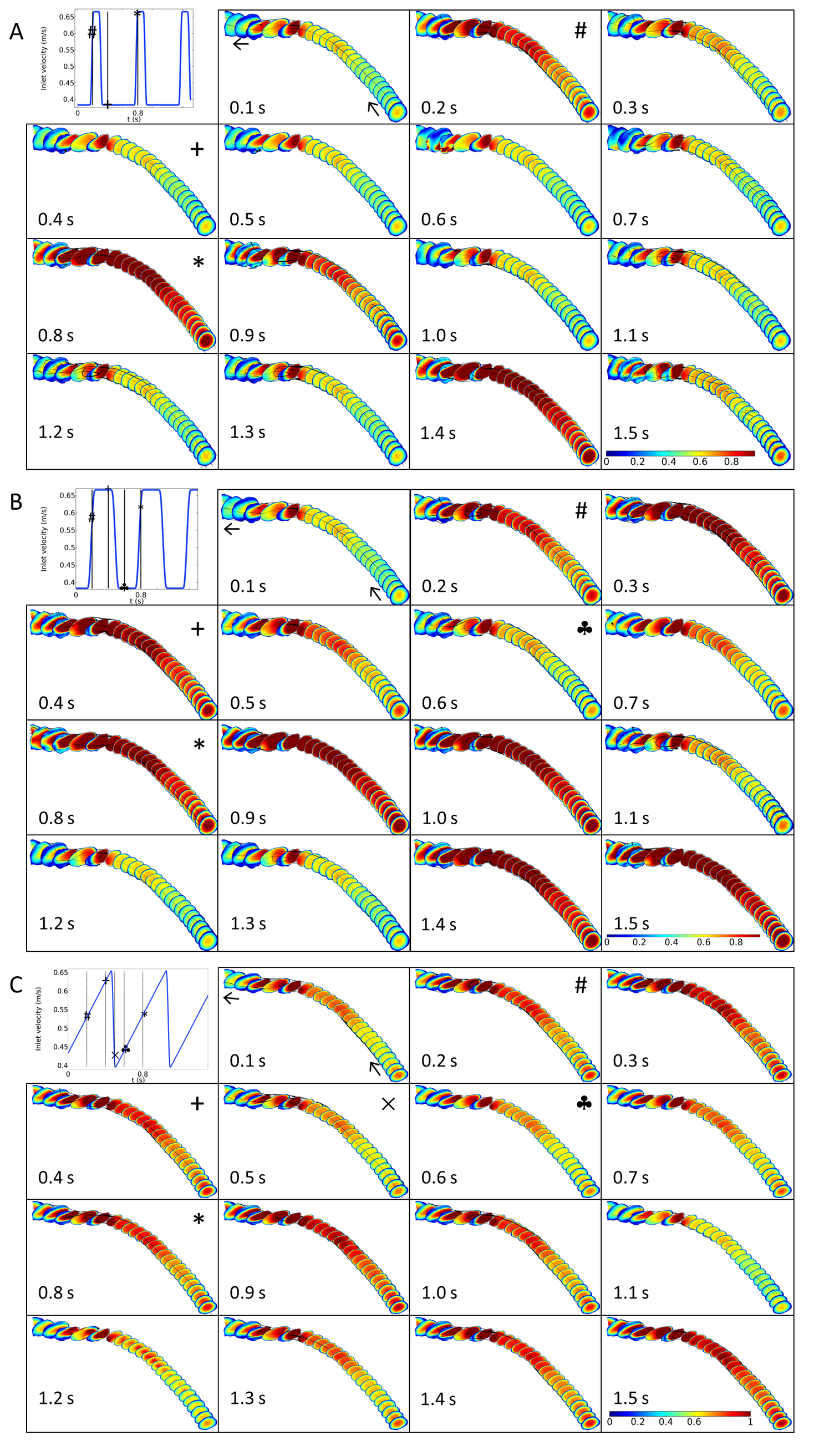

Supplement: S4 Fig — Each subfigure shows the inlet velocity applied as (A) a square wave with duty cycle = 0.5, (B) square wave with duty cycle = 0.2, and (C) a saw-tooth wave. As before, the first panel shows the inlet velocity as it increases (systolic) and decreases (diastolic) as function of time. Inlet velocities and their corresponding velocity profiles are highlighted at three time-points, 0.2, 0.4 and 0.8 s, marked as ‘#’, ‘+’ and ‘*’ in A & B, and four time-points, 0.2, 0.4, 0.5, 0.6 and 0.8 s, marked as ‘#’, ‘+’, ‘×‘, ‘♣‘ and ‘*’ in C. The color bar in the last panel indicates the lowest velocity in blue and the highest in red. Arrows in the second panel indicate the direction of flow along the CA. (TIF) [file pone.0254016.s014.tif]

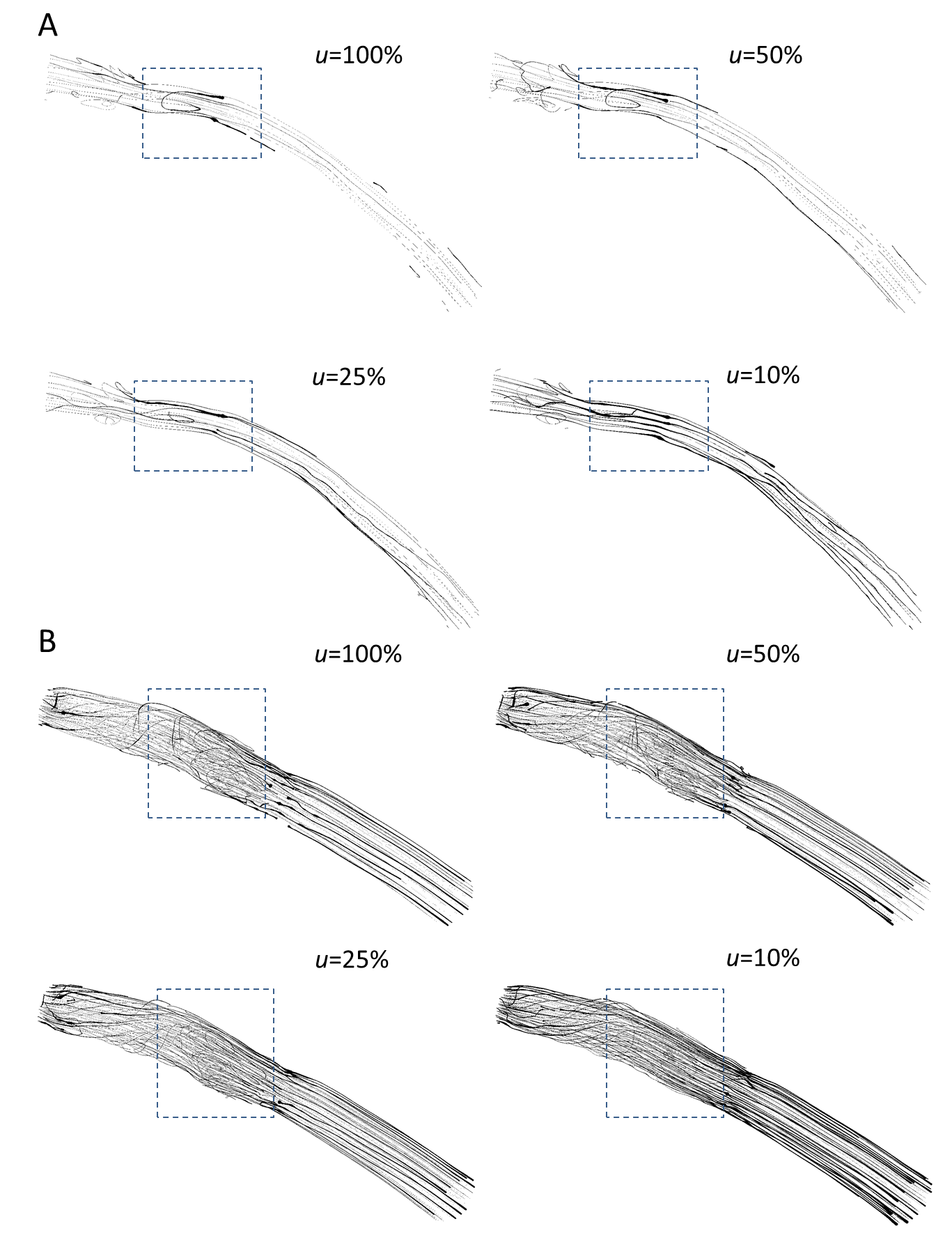

Supplement: S5 Fig — Black tubes indicate the velocity field at that point, with the tube diameter being proportional to the shear rate. Inlet velocities used in simulating flow in each panel are at 100%, 50%, 25% and 10% of the measured systolic velocity, u in Table 2. All other flow parameters are obtained from each patient’s vitals and Doppler data. The ROI for the patient and time-point are highlighted in dashed squares. (A) Patient P96 at 3 mo.; (B) Patient P122 at 12 mo. (TIF) [file pone.0254016.s015.tif]
