# Supplementary material for: Computational modeling of the cephalic arch predicts hemodynamic profiles in patients with brachiocephalic fistula access receiving hemodialysis
Source: PLoS One. 2021 Jul 14;16(7):e0254016. doi: 10.1371/journal.pone.0254016 (PMC8279323; doi:10.1371/journal.pone.0254016)
Supplement: Attachment — Submitted filename: PONE REVIEW Revision Comments Final, Final, Final.docx [file pone.0254016.s017.docx]

April 2, 2021

Dear Editors,

Please find a response to reviewer’s comments below. Our response is in **bold**.

We thank-you for all your time, as these revisions have greatly strengthened the paper.

Mary Hammes

PONE-D-20-36719
A novel computational model of the cephalic arch predicts hemodynamic profiles in patients with brachiocephalic fistulas receiving hemodialysis
PLOS ONE

Reviewer #1: This work highlights patient-specific computational modelling approach to model BC AVF haemodynamic of 5 patients. The study portray the need of patient-specific modelling, however emphasis of outcome predictions haven’t been discussed in detailed in the paper.

**We thank the reviewer for emphasizing the need for more discussion on outcome predictions. We have expanded the text in the *Clinical follow up section* to discuss Figure 5 which shows the flow velocity and pressure profiles at 3 and 12 months with anatomic correlation to cephalic arch and collateral vein formation at a later time points to include the duration of the study which is 5 years.**

“Computational modelling of Cephalic Arch”- this section highlights the geometry image processing of the AVF model, instead of the computational modelling of it, in which the name of this subsection can be misleading here. **We agree and have changed the subsection to “*Image Processing and 3D Reconstruction of the Cephalic Arch”.***

The errors/discrepancies of the image processing approach should be outlined.

**We apologize for the lack of clarity on this point. The most conservative estimate of resolution is 34 µm along the z axis from the IVUS pullback measurements and 32x32 µm^2^ in the xy plane from the resolution of the IVUS images. These numbers have been added to the Methods and Materials section, line 217-219 and 223-224.**

There are too many commercial software specific terms used in the methods and material section (both Computational modelling of cephalic arch and flow simulation). These should be explained using general terms, rather than anything specific to a particular GUI or software. **We thank the reviewer for this comment. We have re-written the Materials and Methods section to simplify the text for the general audience. We also aim to provide a detailed outline of the 3D modeling and flow simulation steps such that the readers can recapitulate the analysis steps using computer aided design software for 3D modeling and finite element analysis software for flow simulation.**

The computational modelling/flow simulation section needs to be further elaborated to explain the validity of this model. Meshing strategy and convergence test needs to be included.

**We thank the reviewer for this comment. We believe that we have established the CA models’ validity (albeit indirectly) as they are consistent between time-points (3 and 12 mo.) for each patient and track well with the clinical outcomes over the period of study. We have also established the validity of the 3D modeling of the CA directly by constructing a fluidic device using 3D printing (please see figure below). Briefly, we printed the 3D model of P104, 3 mo. time-point. We then performed IVUS imaging on the fluidic device. Since our fluidic model was cast from a transparent elastomer, we filled the device with a dye and obtained the general contour of the CA model from optical imaging, in lieu of venogram. We then combined the optical and IVUS images to generate a 3D model using the same procedure as before. The new computational 3D model of the fluidic device thus constructed matched the 3D model of the patient’s CA.**

G

**Figure: Validation of the computationally reconstructed CA models from patient data that are used to construct fluidic devices. A) Fluidic device model of the CA canulated with transducer. B) IVUS catheter C. Portable IVUS counsel D. IVUS image obtained from the mCA device. E) Model reconstructed from IVUS and venogram imaging on patient. F) Model reconstructed from IVUS and optical imaging of mCA device. G) Difference in surface areas between original and reconstructed models from two independent IVUS pull-back measurements as the models are rotated by 180^o^ for comparison.**

**As per reviewer suggestion, we have elaborated on meshing convergence and solver configuration for flow simulation in the Methods and Materials section, line 281-287.**

Pulsatile flow- results on only one patients were outlined. Given the variability of pulsatile flow, would recommend looking into the other patients.

**We thank the reviewer for this comment. We have provided the simulations of pulsatile flow in two additional subjects for 3 mo. and 12 mo. time-points (P93 and P122) as supplementary figures (Figs. S2A&B, S3A&B). We have also included an interpretation of the findings in the Results and Discussion sections. In interest of the manuscript length, we do not provide similar figures for P98 and P104 in the manuscript but provide videos of velocity profiles under pulsatile flow for all 5 subjects as supporting material.**

It is always interesting to link computational CFD studies to clinical observations. The author outlined clinical observations and follow up for the 5 patients. However, the author could draw a better link i.e. how did the CFD results relate to access dysfunctionality observed for the different patients, or in prediction of venous stenosis. **We have expanded the *Clinical Follow up section specifically* linking the CFD results specifically the high velocity profile correlation with both stenosis and collateral vein formation.**

Line 91-94: There were a few key info here but only one references were used. This should be backed up by other literature on the topic. **This is an important statement and additional references [7, 8] were added for support.**

Line 115: There are studies that modelled 3D geometry and flow in ESRD patients at different time points eg. Bozzetto et al. 2016, Carroll et al. 2020 etc. **We have reviewed and included both important references (12, 13). Our work presented in this paper uses a novel approach perform computational modeling from multimodal imaging data, including IVUS, venogram, Doppler, WBV, etc. The computational models highlight marked heterogeneity between patients and time points which makes computational models using patient-averaged data to be non-representative and hard to interpret.**

Line 147 & Line 157: A figure of the geometrical overview of BC AVF, with the CA indicated would have been useful. Confused as to the 10cm proximal to cephalic arch location mentioned here. Also, keep the term used consistent eg. cephalic-axillary vein junction (Line 158) and cephalic arch (Line 148). Line 260-262 & Line 318: Unclear where “pre-bend”, “bend” and “post-bend” were located. **We acknowledge the importance of a visual schematic to convey the intricacy of the CA model downstream of the AVF and have included Figure 1A which shows the anatomy of the brachiocephalic fistula and the cephalic arch with pre-bend, bend and post bend in Figure 1B.**

Line 149: Any particular reason behind the used of 60-degree angle insonation? **The 60 degrees angle of insonation was used to standardize all measurements. References 17 and 18 were added for support.**

Line 228: Ref. should be included for COMSOL Multiphysics

**We thank the reviewer for this suggestion. The reference has been added to the manuscript as follows:[21]: COMSOL Multiphysics® v. 5.5. www.comsol.com. COMSOL AB, Stockholm, Sweden.**

Line 267: Quantify the difference in results between the turbulent closure model and laminar model**We thank the author for this comment. As pointed out by Reviewer 2, turbulent modelling is not strictly necessary for our flow regime (*Re* < 2,000). We have therefore removed any mention of the turbulent model and concentrate solely on the results from the laminar flow modeling in this manuscript for simplicity.**

Line 268-272: This part assumes the waveform to be sinusoidal based on the equation provided. How was pulse frequency obtained? Based on Figure 1, it seems that the flow profile from the Doppler measurement can be non-sinusoidal, has the variability be accounted here?

**We thank the reviewer for this comment. The pulse frequency was calculated from the pulse measured during the Doppler procedure (as number of beats per minute or 60 seconds). We acknowledge that the Doppler waveforms can be non-sinusoidal too. We tested other waveforms (square, saw-tooth) and find that our results remain largely unchanged under these conditions. We have included flow simulations for patient P96 at 3 mo. under square (duty cycle= 0.2 and 0.5) and saw-tooth waveforms as supplementary Figure S4.**

Line 306: Where abouts on the cardiac cycle was the Reynold number calculated (systole or diastole)? **We apologize for the lack of clarity on this. The Reynolds number was calculated in systole. We have made a note of this in Methods and Materials.**

Line 349: Ref. [16] do not seem to be incorrect

**The correct reference here is 14. We apologize about this incorrect reference.**

Line 368-370: This is unclear. Need to be further elaborated with the results obtained. In the discussion section, there are also more factors than just velocity increment that causes the change in Newtonian or non-Newtonian fluid, such as viscosity and shear rate. **We thank the reviewer for this comment. We have elaborated the paragraph to describe the results shown in Fig. 4E-F. We also agree with the reviewer that parameters other than velocity are involved. However, a previous study has shown that under similar conditions, it makes no appreciable difference whether blood is modeled as Newtonian or shear-thinning fluid (see lines 272-274 in the Methods and Materials section).**


Figure 4: x-axis is labelled as diameter, which can be misleading. Clearer description is needed here. **We thank the reviewer for pointing this out and apologize about this confusion. We have relabeled the x-axis as ‘Distance from the center of tube’ where the center is set to zero.**

Figure 5A and 5B: Unsure how the “flow parameters obtained from patients vitals and Doppler etc..” fits in with the figure provided. **We thank the reviewer for this comment. We have revised the legend for Figure 5 and removed the aforementioned sentence.**

Figures can be tidied (Figures 5 and 6 -Velocity contour plots) by removing background grids as they fail to add any value in the understanding of the spatial location of the geometry.

**We thank the reviewer for this comment. We have removed the background grids from all figures containing 3D profiles of velocity, pressure, Re_cell_ and WSS as suggested.**

Figure S1A and S1B: Graphs on Re_cell and WSS were not properly labelled. Unsure which graph represents what. Inconsistent figures location for P104.

**We thank the reviewer for pointing this out. The top profile is for Re_cell and the bottom one is for WSS; we have noted this in the figure legend and text. We have also corrected the ordering of Re_cell and WSS for patient P104 to be consistent with the other patients.**

Table 2 is incomplete- Pulse frequency was not found in the table, which is quite a key information in the study as mentioned under Pulsatile flow section, unless the author intent for Pulse (beat/min) = Pulse frequency? For cases such as P93-3mo., P96-3mo., P98-3mo. and P122-3mo that do not have a pulse (beat/min) noted, the author assumes pulse rate obtained from a different time point to obtain the sinusoidal graph for the inlet boundary conditions. This needs to further justify as it assumes that the pulse rate remains the same for the 3mo. and 12 mo. time point, which may not be the case. **We thank the reviewer for this comment. It is true that the pulse rate of a patient at 3 mo. can be different from the 12 mo. measurement. After reviewing additional data of the cases, we were able to report the missing pulse rate data in Table 2 which has been updated accordingly. We also used the updated information for computational simulations of pulsatile flow.**

Reviewer #2: The authors investigated on five patients the changes in morphology and haemodynamics of the cephalic arch 3 and 12 months after creation of a brachiocephalic fistula. While the manuscript represents a large amount of work, the reviewer fails to identify its clinical relevance nor advancement in medical imaging or engineering that may contribute to a better understanding of the mechanism of haemodynamic alterations after AVF placement. **We have added the clinical relevance to this study of hemodynamics to the discussion. We state that “these computational models lay the foundation for a study to determine and optimize the flow and pressure parameters which could prevent altered hemodynamics that cause AVF complications and failure.** **Using computational data, velocity and pressure could be optimized at the anastomosis by external devices such as Optiflow [36] and VasQ [37] or internal devices such as stents [38] which are designed to minimize flow disturbances with consequences on the CA”.**

The major concern is relating to the aim of the study, which was established to explore the plausible haemodynamic identifiers that may predict stenosis of the AVF, thereby aiding the optimisation of AVF placement. The authors spent a great of time in reconstructing the cephalic arch and obtaining the velocity contours and pressure distributions, whereas their association with AVF patency or stenosis has not been studied nor can be established based only upon 5 patients. Future study should include more subjects and a statistical test of the prognostic performance. **We have stated the aim and hypothesis in the Introduction. “The overall aim of this work is to demonstrate the feasibility of computational modeling to unveil abnormal flow dynamics in the CA that are unique to individual patients”. The importance of future studies was also added to the last paragraph.**

The simulation results, on the other hand, seem intuitive to researchers with a basic background of fluid mechanics — when flow passing through a curved tube, it will yield an elevated pressure on the outer wall of the bend, and possibly a recirculation or flow stagnation zone at the inner side where flow velocity would be lower, as shown in Figures 4 and 5. This phenomenon could be predicted based on the geometry of a flow channel, without the need for a dedicated CFD simulation. **When simulations are done on an idealized model (Figure 4) the results are more intuitive. When simulations are done using actual images from patients, the hemodynamics are not predictable and why we feel the individual approach is important.**

Moreover, as the highest Reynolds number given in Table 2 is only 1,952, there is no reason for the authors to attempt a turbulent model. If divergence happened, the reviewer would suggest refining the computational mesh. **We thank the author for pointing this out. We agree that the turbulent model is not necessary for our flow regime (*Re* < 2,000). We have therefore removed any mention of the turbulent model and concentrate on the results of the laminar model only in this manuscript. As such, we do not encounter any divergence issues in our simulation.**
