# Supplementary material for: Computational modeling of the cephalic arch predicts hemodynamic profiles in patients with brachiocephalic fistula access receiving hemodialysis
Source: PLoS One. 2021 Jul 14;16(7):e0254016. doi: 10.1371/journal.pone.0254016 (PMC8279323; doi:10.1371/journal.pone.0254016)
Supplement: Attachment — Submitted filename: Rebuttel letter Final June 1.docx [file pone.0254016.s018.docx]

PONE-D-21-10937
A novel computational model of the cephalic arch predicts hemodynamic profiles in patients with brachiocephalic fistula access receiving hemodialysis
PLOS ONE

Please include the following items when submitting your revised manuscript:

- A rebuttal letter that responds to each point raised by the academic editor and reviewer(s). You should upload this letter as a separate file labeled 'Response to Reviewers'.

Journal Requirements:

When submitting your revision, we need you to address these additional requirements.

1. Please ensure that your manuscript meets PLOS ONE's style requirements, including those for file naming. The PLOS ONE style templates can be found at

[https://journals.plos.org/plosone/s/file?id=wjVg/PLOSOne_formatting_sample_main_body.pdf](https://mail.uchospitals.edu/owa/redir.aspx?C=zf9stme6SFnCy1DYEa1R1nTTDP3y6o_mI0UBiUAADjrm0yB-RhrZCA..&URL=https%3a%2f%2furldefense.com%2fv3%2f__https%3a%2f%2fjournals.plos.org%2fplosone%2fs%2ffile%3fid%3dwjVg*PLOSOne_formatting_sample_main_body.pdf__%3bLw!!OaZRxjSJGsACmqMyc8s!Y8DzQ47NbjrXghGfzbscoiKBag52U4gD63BMZXVa-uyS4PYqhYUxlYyIC34ny8V2tOBA9tqSs7ii%24) and

[https://journals.plos.org/plosone/s/file?id=ba62/PLOSOne_formatting_sample_title_authors_affiliations.pdf](about:blank)

**Response: We reviewed the PLOS ONE’s style requirements and formatted our manuscript accordingly.**

2.Your ethics statement should only appear in the Methods section of your manuscript. If your ethics statement is written in any section besides the Methods, please delete it from any other section.

**Response: The ethics statement is written in the first paragraph of the Methods section.**

3.Thank you for stating the following in the Acknowledgments Section of your manuscript:

"A. M-R was supported by the NSF GRFP fellowship. The work was partly funded by the Ginny and Simon Aronson Research Award, University of Chicago Institute of Translational Medicine Pilot Award, and A.B.’s research development funds." We note that you have provided funding information that is not currently declared in your Funding Statement. However, funding information should not appear in the Acknowledgments section or other areas of your manuscript. We will only publish funding information present in the Funding Statement section of the online submission form.

Please remove any funding-related text from the manuscript and let us know how you would like to update your Funding Statement. Currently, your Funding Statement reads as follows:

"This publication was made possible by the National Institute of Diabetes and Digestive Diseases (NIDDK) and the National Institutes of Health (NIH) under award number RO1DK090769. "

Please include your amended statements within your cover letter; we will change the online submission form on your behalf.

**Response:** **We have deleted the statement, “A. M-R was supported by the NSF GRFP fellowship. The work was partly funded by the Ginny and Simon Aronson Research Award, University of Chicago Institute of Translational Medicine Pilot Award, and A.B.’s research development funds” from the Acknowledgements and would like this statement to be included in the Funding Statement.**

Additional Editor Comments:

Thank you for resubmitting this work. I have invited a third person to give comments on the revision, who has a few major concerns. I would encourage you to carefully consider these comments, and revise the manuscript for further consideration.


[Note: HTML markup is below. Please do not edit.]

Reviewers' comments:

Reviewer's Responses to Questions

**Comments to the Author**

1. Is the manuscript technically sound, and do the data support the conclusions?

The manuscript must describe a technically sound piece of scientific research with data that supports the conclusions. Experiments must have been conducted rigorously, with appropriate controls, replication, and sample sizes. The conclusions must be drawn appropriately based on the data presented.

Reviewer #1: Partly

**Response: We have added in the discussion the limitations of the small sample size We provide in-depth descriptive, numerical and quantitative analysis. The future work will be to prospectively look at a large cohort of subjects to evaluate the statistical significance of computational modeling to predict clinical outcomes.**

2. Has the statistical analysis been performed appropriately and rigorously?

Reviewer #1: Yes

3. Have the authors made all data underlying the findings in their manuscript fully available?

The [PLOS Data policy](https://mail.uchospitals.edu/owa/redir.aspx?C=jKexNz4yGoe1-wKLRuT6W5qCez7r3MlBR7b5-Mj1hXnm0yB-RhrZCA..&URL=https%3a%2f%2furldefense.com%2fv3%2f__http%3a%2f%2fwww.plosone.org%2fstatic%2fpolicies.action*sharing__%3bIw!!OaZRxjSJGsACmqMyc8s!Y8DzQ47NbjrXghGfzbscoiKBag52U4gD63BMZXVa-uyS4PYqhYUxlYyIC34ny8V2tOBA9kRqqn91%24) requires authors to make all data underlying the findings described in their manuscript fully available without restriction, with rare exception (please refer to the Data Availability Statement in the manuscript PDF file). The data should be provided as part of the manuscript or its supporting information, or deposited to a public repository. For example, in addition to summary statistics, the data points behind means, medians and variance measures should be available. If there are restrictions on publicly sharing data—e.g. participant privacy or use of data from a third party—those must be specified.

Reviewer #1: Yes

4. Is the manuscript presented in an intelligible fashion and written in standard English?

PLOS ONE does not copyedit accepted manuscripts, so the language in submitted articles must be clear, correct, and unambiguous. Any typographical or grammatical errors should be corrected at revision, so please note any specific errors here.

Reviewer #1: Yes

5. Review Comments to the Author

Please use the space provided to explain your answers to the questions above. You may also include additional comments for the author, including concerns about dual publication, research ethics, or publication ethics. (Please upload your review as an attachment if it exceeds 20,000 characters)

Reviewer #1: The overall aim of this work is to demonstrate the feasibility of computational modelling to unveil abnormal flow dynamics in the CA that are unique to individual patients. Five patient-specific models are reconstructed and considered by using commercial software. However, it is not convincing for me to accept it as the innovation as similar research has been conducted before. Even machine learning method has been considered to establish a model for the prediction of a similar hemodynamic problem. The major conclusion from this work is claimed to prove the importance of patient-specific model and the feasibility of using numerical simulation to resolve the flow dynamics, which is not brand new and sounds lacking deeper information. Actually, this point also agrees with the comments of Review 2. The patient-specific numerical simulation has been conducted, but detailed analysis is missing. I suggest the author to do more analysis in terms the change of the stenosis, and possible to provide some insights for the therapy.

**Response: We thank the reviewer for this comment. We agree with these comments and have removed the word ‘novel’ in the title as others have reconstructed hemodynamics using computational software.**

**Previous computational work has been done as discussed in the Introduction with references 9-11, although our work moves the field forward as we use actual images obtained from IVUS and venogram in patient-specific models in 3D with along with computational modeling of flow using parameters from Doppler imaging, blood viscosity measurements, and patients’ vitals recorded at the time of different imaging used in the study. The 5 subjects studied had models created at 3 and 12 months with obvious hemodynamic changes depicted most significantly on the lower border of the bend in the cephalic arch. Follow-up clinical venograms at later time points (up to 5 years) showed significant stenosis with anatomic correlation. We have shown that changes as early as 3 months may be connected to later venous stenosis.**

**The comments provided by the reviewer above have prompted us to discuss potential therapeutic solutions to the future complications, based on our modelling. The hemodynamic changes and resultant wall shear stresses are largely influenced by high flow velocity in the cephalic arch. Our hypothesis is that a reduction in flow velocity will change the turbulent flow toward laminar flow. We tested this by reducing the flow to 50%, 25% and 10% of the original velocities measured in two extreme cases (P96 at 3 months and P122 at 12 months) and have added these results to a new sub-section, ‘*Simulations with decreased flow velocity’* along with a new supplementary figure (Figure S5). This can provide direct insights for therapy as reduced flow (more laminar) may prevent the complications caused by hemodynamic turbulence. Lower flow velocities could be achieved by limiting flow when the BCF is created by 1) limiting the size of the arteriotomy (reference 40 was added); 2) changing the angle of the anastomosis with devices such as a VasQ (reference 36); or 3) the design of the type of anastomosis to promote lower flow (references 38, 39 were added). These insights for the future were included in the discussion.**

6. PLOS authors have the option to publish the peer review history of their article ([what does this mean?](https://mail.uchospitals.edu/owa/redir.aspx?C=KKdvYbaeJDoIi04irp4EKnuR0Zckbjo17xgZSzw5BEHm0yB-RhrZCA..&URL=https%3a%2f%2furldefense.com%2fv3%2f__https%3a%2f%2fjournals.plos.org%2fplosone%2fs%2feditorial-and-peer-review-process*loc-peer-review-history__%3bIw!!OaZRxjSJGsACmqMyc8s!Y8DzQ47NbjrXghGfzbscoiKBag52U4gD63BMZXVa-uyS4PYqhYUxlYyIC34ny8V2tOBA9oB3Xol-%24)). If published, this will include your full peer review and any attached files.


If you choose “no”, your identity will remain anonymous but your review may still be made public.


**Do you want your identity to be public for this peer review?** For information about this choice, including consent withdrawal, please see our [Privacy Policy](https://mail.uchospitals.edu/owa/redir.aspx?C=dSkbbhIMlmEiSdtfinfNyal14CnoOwAQ05bkyKgSIifm0yB-RhrZCA..&URL=https%3a%2f%2furldefense.com%2fv3%2f__https%3a%2f%2fwww.plos.org%2fprivacy-policy__%3b!!OaZRxjSJGsACmqMyc8s!Y8DzQ47NbjrXghGfzbscoiKBag52U4gD63BMZXVa-uyS4PYqhYUxlYyIC34ny8V2tOBA9gcbPyaK%24).

Reviewer #1: No

[NOTE: If reviewer comments were submitted as an attachment file, they will be attached to this email and accessible via the submission site. Please log into your account, locate the manuscript record, and check for the action link "View Attachments". If this link does not appear, there are no attachment files.]

While revising your submission, please upload your figure files to the Preflight Analysis and Conversion Engine (PACE) digital diagnostic tool, [https://pacev2.apexcovantage.com/](https://mail.uchospitals.edu/owa/redir.aspx?C=kKJjrlN-Hyr4hwyZc68JIdIBpUeAMTpjsLdnue2gGULm0yB-RhrZCA..&URL=https%3a%2f%2furldefense.com%2fv3%2f__https%3a%2f%2fpacev2.apexcovantage.com%2f__%3b!!OaZRxjSJGsACmqMyc8s!Y8DzQ47NbjrXghGfzbscoiKBag52U4gD63BMZXVa-uyS4PYqhYUxlYyIC34ny8V2tOBA9nxK-ydA%24). PACE helps ensure that figures meet PLOS requirements. To use PACE, you must first register as a user. Registration is free. Then, login and navigate to the UPLOAD tab, where you will find detailed instructions on how to use the tool. If you encounter any issues or have any questions when using PACE, please email PLOS at [figures@plos.org](https://mail.uchospitals.edu/owa/redir.aspx?C=9Q08397fWhN6HsGfDY7DQdVbEyofztaMmtXZLXXjUYJJNSN-RhrZCA..&URL=mailto%3afigures%40plos.org). Please note that Supporting Information files do not need this step.
